# Synthesis of ZnO Nanorod Film Deposited by Spraying with Application for Flexible Piezoelectric Energy Harvesting Microdevices

**DOI:** 10.3390/s20236759

**Published:** 2020-11-26

**Authors:** Ernesto A. Elvira-Hernández, Jorge Romero-García, Antonio Ledezma-Pérez, Agustín L. Herrera-May, Ernesto Hernández-Hernández, Luis A. Uscanga-González, Víctor A. Jarvio-Cordova, Gilberto Hurtado, Carlos Gallardo-Vega, Arxel de León

**Affiliations:** 1 Micro and Nanotechnology Research Center, Universidad Veracruzana, Boca del Río Veracruz 94294, Mexico; aelvirah@hotmail.com (E.A.E.-H.); leherrera@uv.mx (A.L.H.-M.); 2 Centro de Investigación en Química Aplicada, Saltillo Coahuila 25294, Mexico; jorge.romero@ciqa.edu.mx (J.R.-G.); antonio.ledezma@ciqa.edu.mx (A.L.-P.); ernesto.hernandez@ciqa.edu.mx (E.H.-H.); gilberto.hurtado@ciqa.edu.mx (G.H.); carlos.gallardo@ciqa.edu.mx (C.G.-V.); 3 Maestría en Ingeniería Aplicada, Facultad de Ingeniería de la Construcción y el Hábitat, Universidad Veracruzana, Boca del Río Veracruz 94294, Mexico; 4 Doctorado en Ingeniería, Facultad de Ingeniería Mecánica y Eléctrica, Universidad Veracruzana, Veracruz 94294, Mexico; luscanga98@gmail.com; 5 Instituto Tecnológico de Boca del Río, Boca del Río Veracruz 94294, Mexico; vjarvio@hotmail.com; 6 CONACYT-Centro de Investigación en Química Aplicada, Boulevard Enrique Reyna 140, Saltillo Coahuila 25294, Mexico

**Keywords:** energy harvesting device, flexible substrate, solvothermal method, piezoelectric films, ZnO nanorods

## Abstract

Industry 4.0 and the Internet of Things have significantly increased the use of sensors and electronic products based on flexible substrates, which require electrical energy for their performance. This electrical energy can be supplied by piezoelectric vibrational energy harvesting (pVEH) devices. These devices can convert energy from ambient mechanical excitations into electrical energy. In order to develop, these devices require piezoelectric films fabricated with a simple and low-cost process. In this work, we synthesize ZnO nanorod film by a solvothermal method and deposit by spraying on ITO (indium-tin-oxide)/PET (polyethylene terephthalate) flexible substrate for a pVEH microdevice. The results of the characterization of the ZnO nanorod film using X-ray diffraction (XRD) confirm the typical reflections for this type of nanomaterial (JCPDS 36-145). Based on transmission electron microscopy (TEM) images, the size of the nanorod film is close to 1380 nm, and the average diameter is 221 ± 67 nm. In addition, the morphological characteristics of the ZnO nanorod film are obtained using atomic force microscopy (AFM) tapping images. The pVEH microdevice has a resonant frequency of 37 Hz, a generated voltage and electrical power of 9.12 V and 6.67 μW, respectively, considering a load resistance of 107.7 kΩ and acceleration of 1.5 g. The ZnO nanorod film may be applied to pVEH microdevices with flexible substrates using a low-cost and easy fabrication process.

## 1. Introduction

Piezoelectric energy harvesting devices can convert kinetic energy from ambient mechanical vibrations into electrical energy [1,2,3]. This is due to the piezoelectric properties of the materials used as the main layer of the energy harvesting devices. These devices can significantly increase the generated electrical energy when their piezoelectric layers have longer mechanical deformations. Qin et al. [4] developed piezoelectric ZnO nanowires to convert low-frequency vibration/friction energy into electrical energy. These ZnO nanowires were grown radially around textile fibers. The fabricated nanowires have potential applications for harvesting light, wind and biomechanical energy using fabrics. Malakooti et al. [5] fabricated fiber-based energy harvesters using hybrid composites. These composites were formed by ZnO nanowires grown on the fiber surface of a woven aramid fabric. These harvesters can convert wasted mechanical energy into electrical energy. In addition, this ZnO nanowire film enhanced the elastic modulus and tensile strength of the composites by 34.3% and 18.4%, respectively. Thus, the ZnO nanowire interface incorporated into fabrics can provide energy harvesting and improve the mechanical strength of the structural materials. Briscoe et al. [6] reported a range of testing methods of nanostructured piezoelectric vibrational energy harvesting (pVEH) devices to obtain a better understanding of their power output and electrical behavior. With these testing methods, the electromechanical behavior of nanostructured pVEH devices can be compared to other energy harvesting methods for future applications. Li et al. [7] presented a fiber-based hybrid energy harvesting device composed of central ZnO nanorods on carbon fibers and double-sided Cu electrodes on nylon film. The ZnO nanorods and nylon film allow this device to convert the biomechanical energy into electrical energy using transduction mechanisms such as piezoelectric and triboelectric. This flexible device has potential applications for smart clothes.

Other researchers [8,9,10,11,12,13] have developed piezoelectric energy harvesting devices using flexible substrates. Lu et al. [8] reported a recent review of polyvinylidene fluoride (PVDF)-based energy harvesting devices, including their structural design, fabrication process, main advantages, challenges, and future applications. These devices can be used to supply electrical energy of flexible intelligent electronics, with low power consumption for smart and flexible wearable devices, to medical monitoring and industrial applications. Koç et al. [9] developed a piezoelectric energy harvesting device composed of PVDF/PZT (lead zirconate titanate) electrospun nanofibers. This harvesting device provides a potential clean energy source from mechanical vibrations for powering portable microsensors. In addition, piezoelectric energy harvesting devices with flexible substrates can be adjusted to different parts of the human body to convert the biomechanical energy to electrical energy. However, more investigations about simple and low-cost fabrication processes of piezoelectric films deposited on flexible substrates must be developed. Here, we synthetize ZnO nanorod film by a solvothermal method for flexible pVEH microdevices. The ZnO film and the top silver electrode of the pVEH device were deposited on ITO (indium-tin-oxide)/PET (polyethylene terephthalate) flexible and lightweight substrate by a spraying method. This ZnO film was deposited at low temperature without high vacuum and expensive equipment. The spraying method does not require high-cost equipment and high technology to synthesize ZnO films when compared with those epitaxially grown, chemical vapor deposition, and electrodeposition. Spraying is a simple method where the material solution can be applied on a wide range of surfaces, including flexible substrates. The use of ZnO nanorods permits low charge recombination. On the other hand, bulk ZnO can form aggregates and giant structures that increase the charge recombination and decrease the microdevice performance. The novelty of this research is to deposit uniform film of ZnO nanorods on a large area of flexible substrate at low temperature using a low-cost process to develop pVEH microdevices. The size of the ZnO nanorod film is measured using transmission electron microscopy (TEM) images, and the ZnO nanorods’ morphological characteristics are determined using atomic force microscopy (AFM) tapping images. The use of flexible matrices in the case of polymers like PVDF, poly(vinylidene fluoride-trifluoroethylene-chlorofluoroethylene) (PVDF-TrFE-CFE) and (chlorotrifluoroethylene) CTFE or their derivates with ZnO nanorods could increase the energy harvesting microdevice efficiency and lifetime. In addition, it could decrease the formation of aggregates, and could allow thousand cycles of mechanical loading on the microdevices.

## 2. Materials and Methods

### 2.1. Materials

Ethylene glycol (solvent 99.8%), ethanol (solvent 99.9%) and isopropyl alcohol (solvent 99.9%) were purchased from J.T. Baker; zinc dehydrate acetate (98%) and ITO/PET substrates were acquired from Sigma-Aldrich Chemical Company, and silver print conductive paint was obtained from GC electronics.

### 2.2. Synthesis of ZnO Nanorod Film

The ZnO nanorod film was synthesized by a modified solvothermal method [13] in a beaker 500 mg of zinc dehydrate acetate was dissolved in a mixture of 10 mL of ethylene glycol and 3 mL of deionized water. This mixture was placed in magnetic stirring for 30 min until the zinc acetate completely dissolved. After this, the mixture was put into a Teflon-lined stainless-steel autoclave (DELTA REACTORY, Saltillo, Coahuila, Mexico) and left to react for 8 h at 150 °C. The product was centrifuged and washed with ethanol three times at 14,000 rpm for 25 min. In the following, the outcome of this reaction will be referred to as ZnO.

### 2.3. Characterization

The X-ray diffraction (XRD) study of ZnO nanorod film was performed on an Eco D8 (Bruker, Billerica, MA, USA) Advance of Bruker. The data were recorded in the range of 3–90° 2θ, at a rate 0.01°/min, 40 KV voltage and 25 mA of emission current. The absorption spectrum was determined by UV-vis spectroscopy with a Shimadzu 2401PC (Shimadzu corporation, Kyoto, Japan) spectrophotometer, using isopropyl alcohol as the reference. The morphology of the ZnO nanorod film was determined by transmission electron microscopy (TEM) with a FEI Titan microscope (FEI company, Hillsboro, OR, USA) at 300 KV, by casting a few drops of dispersion materials in water (0.1 mg/mL) on a Lacey Carbon grid. The samples’ surface morphological characteristics were carried out by atomic force microscopy (AFM) on a Dimension™3100 from Digital Instruments with a Pt-coated Si tip with a 15 nm nominal radius model (OSCM-PT Bruker). The images were obtained in the tapping and deflection mode at a scanning rate of 1.0 Hz for 256 lines. The hysteresis loop was realized using a radiant precision multiferroic (model P-PMF) Vmax 200 V with an area sample of 384.84 × 10^−3^ cm^2^ and a thickness of 1700 μm.

### 2.4. Microdevice Fabrication

The PET-ITO substrate was cleaned in an ultrasound bath with ethanol three times for 10 min and dried at 60 °C in a thermo scientific oven for 30 min. Then, 50 mg of ZnO was dispersed in 5 mL isopropyl alcohol, and the solution was sonicated for 1 h and kept under magnetic stirring for 24 h. The solution of ZnO was used for thin film and was deposited by spraying at 80 °C using an atomizer. To obtain a film of 1500 nm in the top electrode, Ag colloidal solution was used and deposited by spraying at 80 °C. Finally, the silicon seismic mass was added on the pVEH microdevice free side.

### 2.5. Microdevice Performance

**Figure 1:**
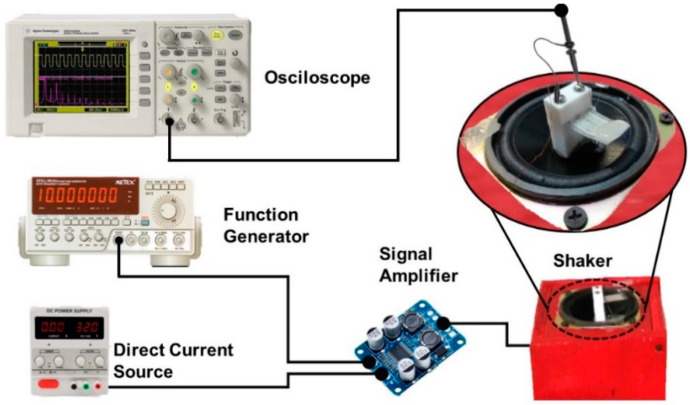
Schematic view of the experimental setup to measure the output voltage of the piezoelectric vibrational energy harvesting (pVEH) microdevice.

The pVEH microdevice was mounted in the central area of an electromagnetic shaker constructed with a woofer coupled (CIQA, Saltillo, Coahuila, Mexico) to a signal amplifier TPA3118 (Texas instruments, Dallas, TX, USA) (Figure 1). The vibration amplitude and frequency of the shaker was obtained using a function generator (METEX model Mxg-9810a) (METEX, Seoul, Korea). This function generator supplied electrical signals with a specific amplitude, frequency and shape, which was amplified. To measure the output voltage of the pVEH microdevice, an oscilloscope (KEYSIGHT Technologies model DSO3102A) (KEYSIGHT Technologies, Santa Rosa, CA, USA) was connected to its upper and lower electrodes.

## 3. Results and Discussion

**Figure 2:**
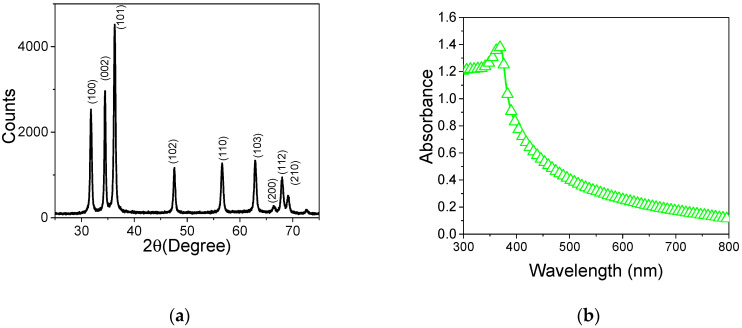
(**a**) XRD diffractograms of ZnO nanorod film and (**b**) absorption spectra of ZnO nanorod film.

The XRD diffractogram of the ZnO nanorod shows the reflections matching the crystalline planes of the material structure: (100), (002), (101), (102), (110), (103), (200), (112) and (201), which are presented in the diffractions at 2θ = (31°), (34°), (36°), (47°), (56°), (62°), (66°), (67°), (69°). These reflections correspond to the crystal structure of hexagonal wurtzite ZnO (JCPDS 36–145). This result demonstrates the total conversion of the precursor (zinc acetate) into ZnO without signals or other associated peaks for impurities (Figure 2a).

The absorption spectra of ZnO exhibit a peak with a maximum at 350 nm typically associated with the absorption of zinc oxide. This peak shows a hypochromic displacement in regard to the bulk material (400 nm) that can be attributed to the nanometric size of the ZnO nanorod film, indicating 3.16 eV for Eg [14] (see Figure 2b).

**Figure 3:**
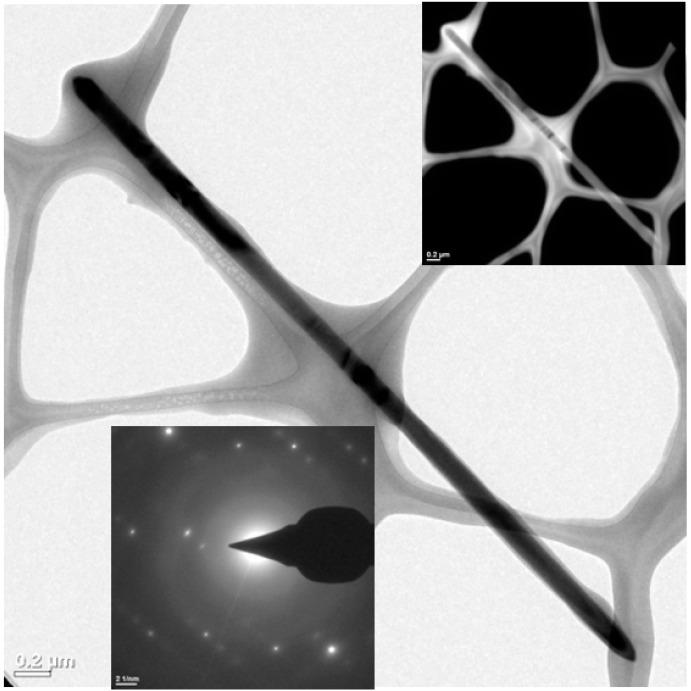
TEM images of an isolated ZnO nanorod.

TEM images (Figure 3) confirm the ZnO nanorods shape and crystalline order with an average size of 1380 ± 200 nm in length and 220 ± 67 nm in diameter. These results agree well with the results obtained by UV-vis spectroscopy and XRD techniques. The ZnO nanorod in the image has a length of 4428 nm and a diameter of 123 nm. The dark-field image (inset in Figure 3) indicates the existence of only one material, which translates into the same color type with similar density in the nanorod.

**Figure 4:**
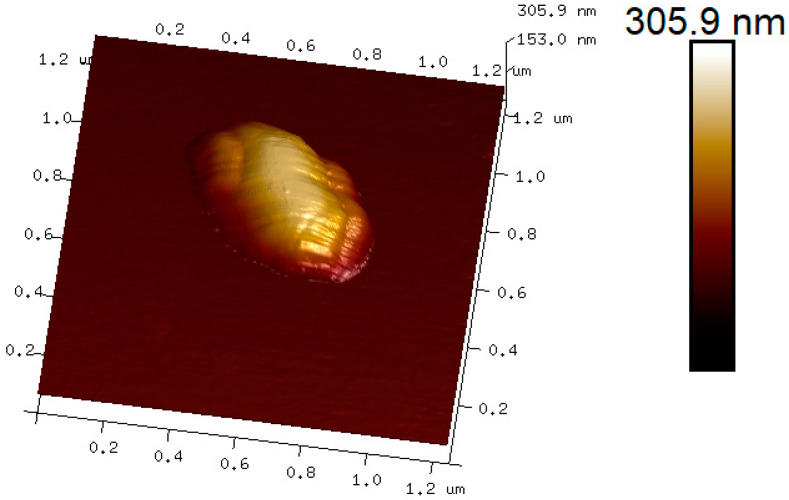
Side view of a morphological image by atomic force microscopy (AFM) for a single ZnO nanorod.

Another way to review the morphological characteristics of the ZnO nanorod is through an AFM tapping image (Figure 4), which confirms a typical ZnO nanorod shape supported by the side-view image. In this case, the diameter of the ZnO nanorod measures close to 300 nm, which has a reasonable proximity with the value obtained from the TEM images.

**Figure 5:**
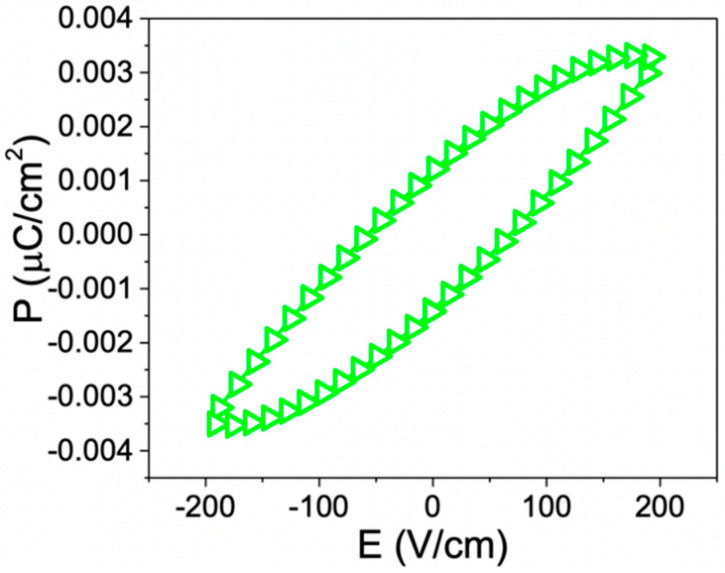
Polarization-electric field hysteresis loops of ZnO nanorod film.

Piezoelectric materials are a subset of ferroelectric materials, where there is a relationship between mechanical stress and voltage. Due to the stress-electric field coupling, piezoelectric materials have been using in a variety of applications. In addition, ZnO nanorod film must be polarized after interacting with an external electric field. Following the above, it was possible to obtain a polarization-electric field (P-E) hysteresis loop for a simple ZnO nanorod film and exhibit a 62.22 V/cm coercive field, 0.00129 μC/cm^2^ remanent polarization and 0.0034 μC/cm^2^ saturation polarization (Figure 5). These results confirm the piezoelectric nature of the ZnO nanorods synthesized in this work.

### 3.1. Electromechanical Behavior

**Figure 6:**
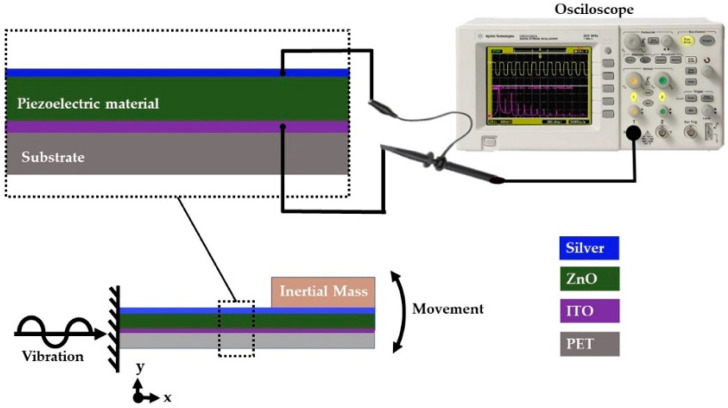
Schematic view of the open voltage (Voc) characterization system of the flexible pVEH microdevice.

**Figure 7:**
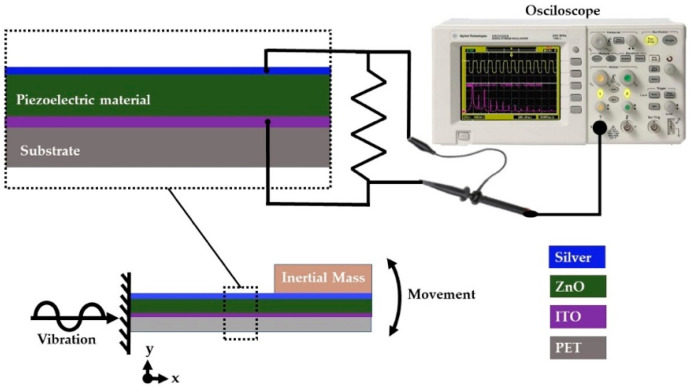
Schematic view of the short circuit (Vsc) characterization system of the flexible pVEH microdevice.

**Figure 8:**
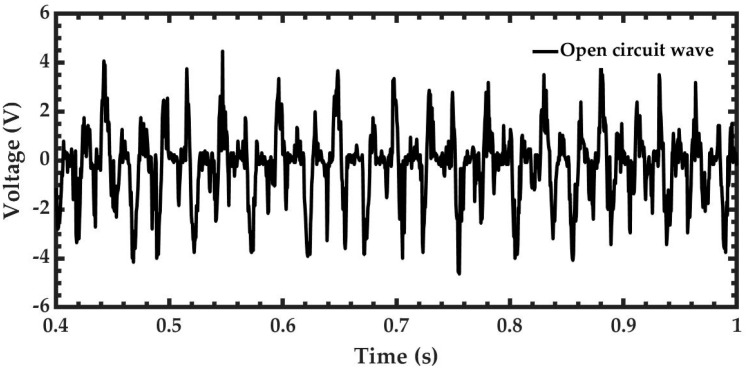
Output voltage of the pVEH microdevice under mechanical vibrations at 37 Hz and 1.5 g acceleration.

The electromechanical behavior of the pVEH microdevice is obtained using two measurements of its output voltage (see Figure 6 and Figure 7): open voltage (Voc) and short circuit (Vsc). The Voc is measured by applying vibrational mechanical force at 37 Hz and 1.5 g of acceleration (14.715 m/s^2^), as shown in Figure 8. The peak-to-peak value of voltage is 9.12 V, and the average voltage is 1.47 Vrms. The supplementary material of the manuscript shows two videos of the electromechanical behavior of the pVEH microdevice under static and dynamic loads.

The mechanism used to generate electric power of the pVEH microdevice is given from the mechanical stress produced through vibration. This provides a displacement for Zn^+2^ cations in relation with the O^2−^ anions inside the ZnO nanorod film in the wurtzite structure. These electrical charges can be harvested by the electrodes of the pVEH microdevice [15].

To measure the Vsc of the pVEH microdevice, a charge resistance was added between its electrodes. In this case, different charge resistances were used to calculate the generated power by the pVEH microdevice (Table 1).

**Figure 9:**
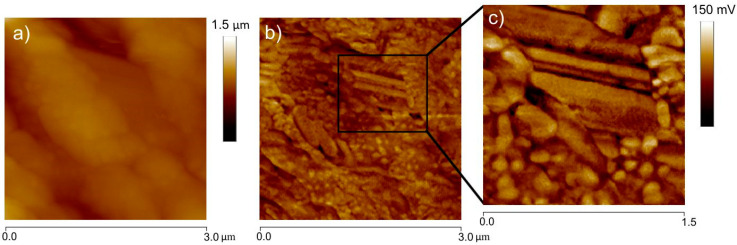
Morphological characteristics (**a**) tapping image and (**b**) deflection image of ZnO nanorod film on ITO (indium-tin-oxide)/PET (polyethylene terephthalate) substrate, (**c**) inset 1.5 μm of deflection image.

After depositing the ZnO nanorod film on ITO/PET substrate, it was possible to clearly identify the nanorod arrangement on the films deposited by the spraying method (Figure 9c). There was a step before depositing the top electrode to conclude with the pVEH microdevice manufacture process. The morphological analysis of the ZnO nanorod film using AFM deflection mode shows that the ZnO nanorods do not have the (001) plane orientation. This depicts a horizontal position for the ZnO nanorods. Thus, the spraying method is a good low-cost alternative for depositing ZnO nanorod films on flexible substrates of pVEH microdevices. With the spraying method, these microdevices will achieve output powers less than those obtained using ZnO nanorods oriented in the plane (001).

### 3.2. Modeling of the pVEH Microdevice

This section presents the mechanical analysis of pVEH microdevice. It shows the resonant frequency, vibrational modes, static displacement and generated stress obtained using finite element method (FEM) models.

#### The Finite Element Method (FEM)

The ANSYS^®^ software was used to obtain the finite element method (FEM) models of the pVEH microdevice. Table 2 shows the mechanical properties of materials considered in these FEM models. With these FEM modes, the first four resonant frequencies and vibrational modes, the static displacement and mechanical stress of the microdevice were determined. A fine mesh was obtained close to the fixed side of the microdevice, where the highest mechanical stresses are concentrated.

**Figure 10:**
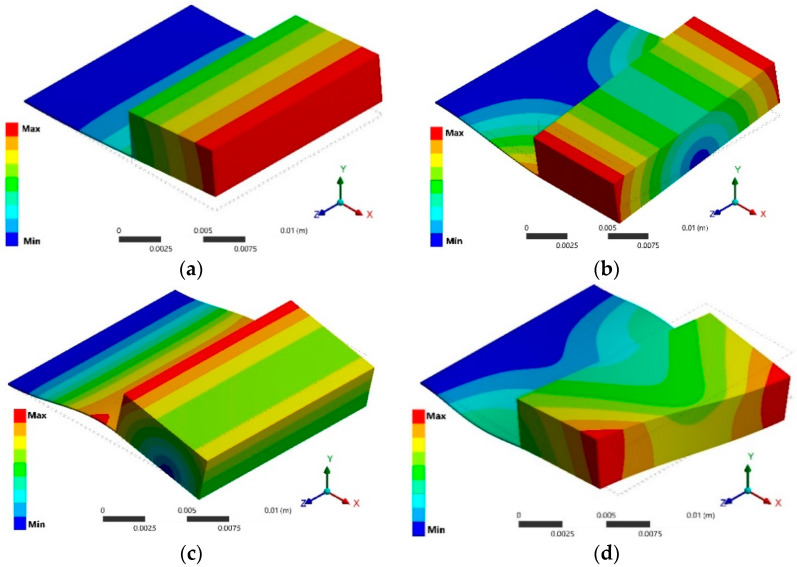
First four vibrations modes of the FEM model of the pVEH microdevice: (**a**) first (38.5 Hz), (**b**) second (142.87 Hz), (**c**) third (371.65 Hz) and (**d**) fourth (2738.7 Hz) vibration mode.

The modal analysis determined the first four vibration modes of the pVEH microdevice. The first out-plane bending vibration mode has a resonant frequency at 38.5 Hz (Figure 10a), with an estimated error of 4.05% with respect to that obtained by the experimental test. The second, third and fourth vibrations modes (see Figure 10b–d) have frequencies of 142.87, 371.65 and 2738.7 Hz, respectively.

**Figure 11:**
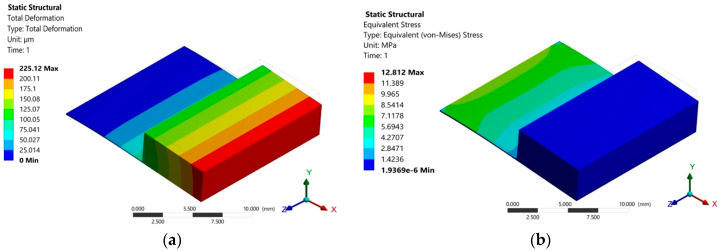
(**a**) Maximum static displacement and (**b**) maximum static stress of the FEM models of the pVEH microdevice caused by the weight of the seismic mass.

**Figure 12:**
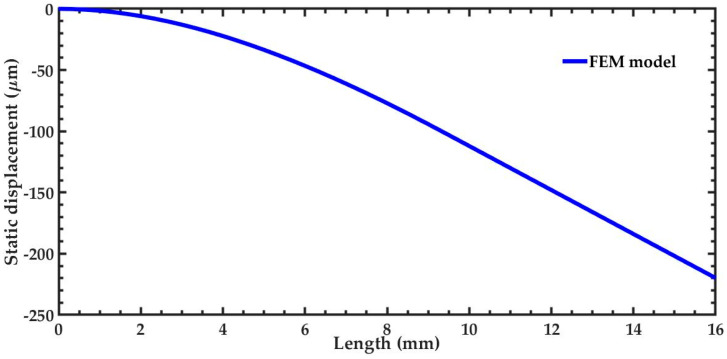
Static displacement of the pVEH microdevice as a function of its length.

The effect of the silicon seismic mass on the structural behavior of the pVEH microdevice was determined through a structural analysis using FEM models. This static structural analysis allows the estimation of the static deflection and the maximum stress of the microdevice. For this analysis, the pVEH microdevice is fixed along its first end, and gravity acceleration (9.81 m/s^2^) is applied in the negative direction of the *y*-axis. Based on this structural analysis, the silicon seismic mass generates a maximum static displacement of 225.12 μm and a maximum stress of 12.812 MPa (Figure 11a,b). Furthermore, the response of the static displacement of the microdevice as function of its length is shown in Figure 12.

A harmonic response analysis of the mechanical behavior of the pVEH microdevice was obtained using FEM models and considering different accelerations (0.2 m/s^2^, 0.4 m/s^2^, 0.6 m/s^2^, 0.8 m/s^2^ and 1.0 m/s^2^) in direction of the *y*-axis. Small acceleration values were selected to match those of environment vibrations. This harmonic response analysis included air damping at atmospheric pressure on the microdevice. This damping can be determined with the quality factor (*Q*) using Blom’s model [16]:

(1) $$Q_{a}=\frac{f_{r}\rho_{p}bhL}{3\mu R\left(1+R/\beta \right)}$$

with

(2) $$\beta =\sqrt{\frac{\mu }{\pi \rho_{a}f_{r}}}$$

(3) $$R=\sqrt{\frac{bL}{\pi }}$$

where *b* is the width, *h* the thickness of the substrate, *L* is the length of the microdevice, *f_r_* is the resonant frequency of the microdevice, *ρ_p_* is the PET density, and *µ* and *ρ_a_* the viscosity and density of the air, respectively. The damping ration (*ζ*) of the pVEH microdevice can be determined by

(4) $$\zeta =\frac{1}{2Q}$$

**Figure 13:**
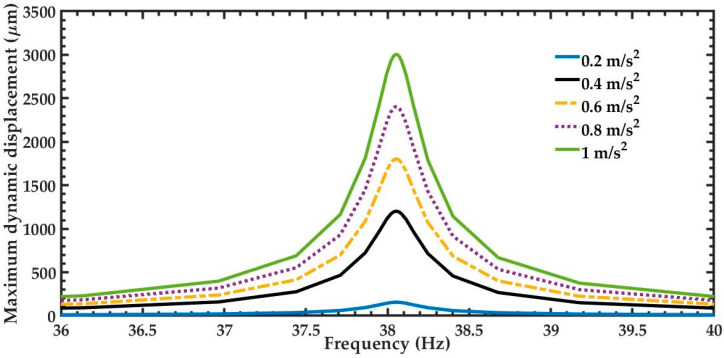
Dynamic displacement of the pVEH microdevice for different acceleration values.

By using Equations (1) and (4), the quality factor and damping ratio of the pVEH microdevice are 132.51 and 37.73 × 10^−4^, respectively. Figure 13 depicts the maximum dynamic displacements of 156, 1201.6, 1802.3, 2403.1 and 3003.9 μm under accelerations of 0.2, 0.4, 0.6, 0.8 and 1.0 m/s^2^, respectively. These maximum displacements are obtained in the free end of the microdevice.

**Figure 14:**
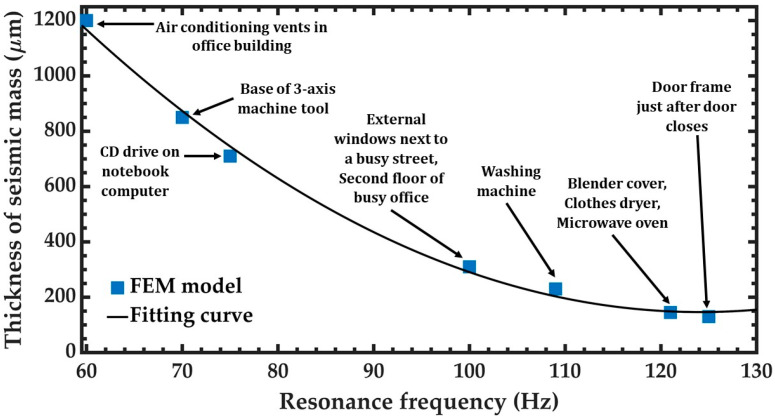
Schematic view of the variation of the resonant frequency of the pVEH microdevice caused by the modification of the thickness of seismic mass. Different applications of the pVEH microdevice can be obtained adjusting the thickness of seismic mass.

The resonance frequency of the pVEH microdevice can be adjusted for matching with that of different mechanical vibration sources. For this, the thickness of seismic mass can be modified to obtain variations in the resonance frequency of the microdevice (Figure 14). Thus, the microdevice can operate at resonance, achieving higher displacements and generated electrical power. With this process, the pVEH microdevice can be designed to operate in specific applications that have different frequencies of mechanical vibrations.

Research of pVEH microdevices has rapidly advanced in the last two decades due to their potential application to supply electrical energy to portable devices. However, these microdevices have challenges such as electric fatigue [17], resulting in reduced switching polarization after a few cycles of operation, and the brittle nature [18] of the ceramic piezoelectric materials (e.g., PZT). Therefore, the purpose of this work was to develop an alternative synthesis (solvothermal) and low-cost fabrication based on a spraying technique for the films deposition on flexible pVEH microdevices. With the flexible PET substrate, the microdevice can achieve high deformations and high stress on the nanostructured piezoelectric film. Thus, the pVEH microdevice can increase its open circuit output voltage. The proposed fabrication method of ZnO nanorod film on ITO/PET substrate can permit the development of low-cost flexible pVEH microdevices.

## 4. Conclusions

A simple and low-cost fabrication process of ZnO nanorod film for potential application in flexible pVEH microdevices is presented. The ZnO nanorod film was synthetized by the solvothermal method and deposited by spraying on ITO/PET substrate. An XRD diffractogram of ZnO nanorod film was obtained showing reflections matching the crystal structure of hexagonal wurtzite ZnO. Furthermore, the size and morphological characteristics of the ZnO nanorod film were measured through TEM and AFM images, respectively. Based on the experimental results, the proposed fabrication method of ZnO nanorod film can be another option with respect to conventional methods to develop flexible pVEH microdevices. Thus, these microdevices can decrease their fabrication costs, keeping suitable electromechanical performance to supply electrical energy to low-power consumption devices.

## Figures and Tables

**Table 1:** Electrical response of the flexible pVEH microdevice with ZnO nanorod film.

Resistance (kΩ)	Voltage Peak to Peak (V)	RMS Voltage (V)	Current (μA)	Power (μW)
107.7	1.2	0.848	7.87	6.67
205.8	1.6	1.131	5.49	6.2
304.7	1.84	1.3	4.26	5.53
761	2.7	1.9	2.49	4.73

**Table 2:** Mechanical properties of materials used in the FEM models of pVEH microdevice.

Material	Density (kg/m^3^)	Young’s Modulus (GPa)	Poisson Ratio
PET	1400	2.4	0.36
Silver	10,490	74	0.395
ZnO	5665	137	0.25
Silicone	1100	0.05	0.49

## Supplementary Materials

The following are available online at https://www.mdpi.com/1424-8220/20/23/6759/s1, Video 1. shows the output voltage of the microdevice when a static load is applied to its free end. Video 1. depicts the output voltage of the microdevice when the shaker is excited with acceleration of 1.5 g and frequency of 37 Hz.
