# Supplementary material for: Synthesis of ZnO Nanorod Film Deposited by Spraying with Application for Flexible Piezoelectric Energy Harvesting Microdevices
Source: Sensors (Basel). 2020 Nov 26;20(23):6759. doi: 10.3390/s20236759 (PMC7729474; doi:10.3390/s20236759)
Supplement: Supplementary file 1 [file sensors-20-06759-s001.zip › Videos description.docx]

**Supplementary Material**

This supplementary material shows the experimental setup to measure the output voltage of the pVEH microdevice. Figure 1 depicts the pVEH microdevice mounted in the central area of an electromagnetic shaker, which was constructed with a woofer coupled to a signal amplifier. This shaker can generate mechanical vibrations on the microdevice. A function generator provides electrical signals with a specific amplitude, frequency and shape, which were amplified. To measure the output voltage of the microdevice, an oscilloscope was connected to its upper and lower electrodes. Two videos of the electromechanical behavior of the pVEH microdevice are included as Supplementary Materials. The first video shows the output voltage of the microdevice when a static load is applied to its free end. The second video depicts the output voltage of the microdevice when the shaker is excited with acceleration of 1.5 g and frequency of 37 Hz.


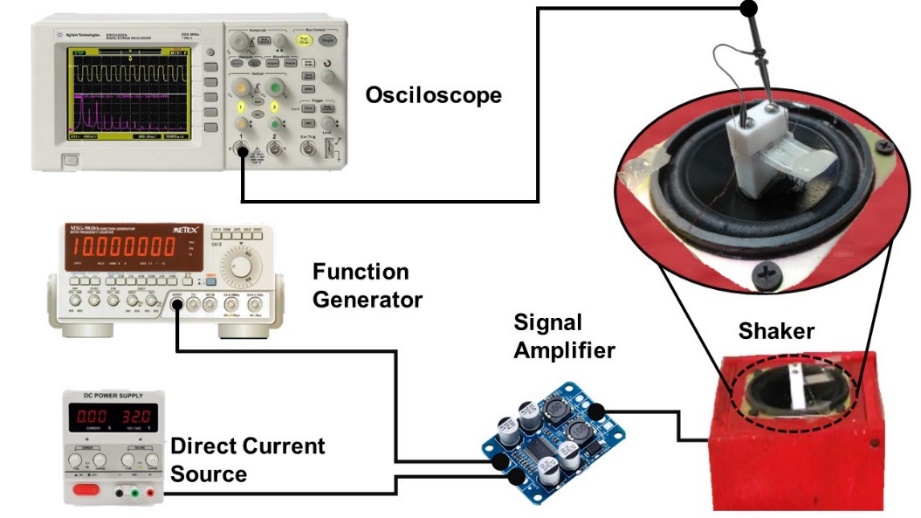


**Figure 1.** Schematic view of the experimental setup to measure the output voltage of the pVEH microdevice.
